# Evaluation of antiviral activity of *Ocimum sanctum* and *Acacia arabica* leaves extracts against H9N2 virus using embryonated chicken egg model

**DOI:** 10.1186/s12906-018-2238-1

**Published:** 2018-06-05

**Authors:** S. S. Ghoke, R. Sood, N. Kumar, A. K. Pateriya, S. Bhatia, A. Mishra, R. Dixit, V. K. Singh, D. N. Desai, D. D. Kulkarni, U. Dimri, V. P. Singh

**Affiliations:** 1 National Institute of High Security Animal Diseases, Anand Nagar, Bhopal, Madhya Pradesh India; 2 Indian Veterinary Research Institute, Izatnagar, Bareilly, Uttar Pradesh India; 3 Present Address: Department of Veterinary Epidemiology and Preventive Medicine, COVAS, Udgir, Latur, Maharashtra India

**Keywords:** *O. sanctum*, *A. arabica*, Avian influenza H9N2, *in ovo* testing, HA, Real time RT-qPCR

## Abstract

**Background:**

In the view of endemic avian influenza H9N2 infection in poultry, its zoonotic potential and emergence of antiviral resistance, two herbal plants, *Ocimum sanctum* and *Acacia arabica*, which are easily available throughout various geographical locations in India were taken up to study their antiviral activity against H9N2 virus. We evaluated antiviral efficacy of three different extracts each from leaves of *O. sanctum* (crude extract, terpenoid and polyphenol) and *A. arabica* (crude extract, flavonoid and polyphenol) against H9N2 virus using *in ovo* model.

**Methods:**

The antiviral efficacy of different leaves extracts was systematically studied in three experimental protocols viz. virucidal (dose-dependent), therapeutic (time-dependent) and prophylactic (dose-dependent) activity employing *in ovo* model. The maximum non-toxic concentration of each herbal extracts of *O. sanctum* and *A. arabica* in the specific pathogen free embryonated chicken eggs was estimated and their antiviral efficacy was determined in terms of reduction in viral titres, measured by Haemagglutination (HA) and real time quantitative reverse transcription polymerase chain reaction (RT-qPCR) assays.

**Results:**

All the extracts of *O. sanctum* (crude extract, terpenoid and polyphenol) and *A. arabica* (crude extract, flavonoid and polyphenol) showed significant virucidal activity, however, crude extract_*ocimum*_ and terpenoid_*ocimum*_ showed highly significant to significant (*p* < 0.001–0.01) decrease in virus genome copy numbers with lowest dose tested. Similarly, therapeutic effect was observed in all three extracts of *O. sanctum* in comparison to the virus control, nevertheless, crude extract_*ocimum*_ and terpenoid_*ocimum*_ maintained this effect for longer period of time (up to 72 h post-incubation). None of the leaves extracts of *A. arabica* had therapeutic effect at 24 and 48 h post-incubation, however, only the crude extract_*acacia*_ and polyphenol_*acacia*_ showed delayed therapeutic effect (72 h post-inoculation). Prophylactic potential was observed in polyphenol_*acacia*_ with highly significant antiviral activity compared to virus control (*p* < 0.001).

**Conclusions:**

The crude extract and terpenoid isolated from the leaves of *O. sanctum* and polyphenol from *A. arabica* has shown promising antiviral properties against H9N2 virus. Future investigations are necessary to formulate combinations of these compounds for the broader antiviral activity against H9N2 viruses and evaluate them in chickens.

## Background

Influenza A viruses (IAVs), members of the family *Orthomyxoviridae* are characterized by a single stranded, segmented negative-sense RNA genome. Among the IAVs, avian influenza (AI) H9N2 has become endemic in terrestrial poultry in several countries of the Eurasian continent including India in recent years [1–4]. The spread of AI H9N2 has resulted in significant economic losses in poultry mainly because of reduced egg production and high mortality associated with co-infection with other respiratory pathogens [5]. Although AI H9N2 does not fall under the definition of highly pathogenic avian influenza (HPAI) viruses, there has been ever increasing speculation about pandemic potential of H9N2 viruses [6].

So far, a total of 28 laboratory-confirmed cases of human infection with avian influenza A (H9N2) viruses, however none fatal, have been detected globally (http://www.who.int/influenza/human_animal_interface/Influenza_Summary_IRA_HA_interface_25_02_2016.pdf). Among them, the recent one includes from China [7]. In addition, serological evidences of AI H9N2 virus exposure to human have been reported on several occasions from Iran, China and India [8–11]. The higher human infection capability of these viruses was provided by the fact that H9N2 binds to α-2,6 sialic acid receptors that are abundant in the human upper respiratory tract while H5N1 chiefly bind to human receptors in the lower respiratory tract [12]. The recently emerged influenza A (H7N9) and (H10N8) infecting humans had acquired gene segments from H9N2 virus [13, 14]. The potential of genetic reassortment of IAVs, due to segmented genome, from different animal species is thought to be a mechanism for the emergence of influenza viruses with pandemic potential [14]. Endemicity of H9N2 circulation in poultry especially in India could further aggravate the current situation. Among the control measures, existing vaccines are unable to keep up with the mutation rates of viruses. New vaccine development takes a long time and at the same time, viruses are also developing resistance to the currently used drugs [15]. Hence, there is no immediate response drug to the newly emerging virus infections/outbreaks. To address this problem, there is an exigent need for the development of a new paradigm preventive and therapeutic agent to control the immediate spread of viral outbreaks. In this scenario, traditional herbal medicines have been postulated to prove effective due to fewer side effects, relatively low cost and easy availability [16].

Two plants, *Ocimum sanctum* and *Acacia arabica* are widely distributed and easily available throughout various geographical locations in India. The efficacy of *O. sanctum* as inhibitory compound has been documented against several viruses like Newcastle Disease virus, Vaccinia virus and Infectious Bursal Disease virus [17]. Similarly, *A. arabica* has been explored for its virucidal properties against Peste des petits ruminants (PPR) virus [18], along with inhibition of Goatpox virus replication [19]. However, antiviral H9N2 influenza activity of different extracts derived from the leaves of these two plants has not been studied.

For evaluation of the antiviral properties of medicinal plants against IAVs, three methods viz. tissue culture [20, 21], laboratory/experimental animals [22] and *in ovo* model [23] have been used frequently. Each method has its pros and cons. We preferred *in ovo* model, which is at the borderline of in vitro and in vivo studies and thus does not conflict with either ethical or legal aspects of animal protection [23, 24]. Therefore, keeping in view the endemicity and pandemic potential of H9N2, this study was taken up to assess the H9N2 inhibitory potential of various extracts derived from leaves of *O. sanctum* and *A. arabica* using *in ovo* model.

## Methods

### Virus and embryonated chicken eggs

Avian Influenza virus, A/chicken/CL/15–12/103075 (H9N2) was collected from one of the major water body of Maharashtra state of India during an ongoing surveillance programme. Specific pathogen free (SPF) embryonated chicken eggs (ECEs) were obtained from SPF Unit of ICAR- National Institute of High Security Animal Diseases, Bhopal, India. This virus was passaged two times in the allantoic fluid of 10-days-old SPF ECEs to make seed virus stock. The virus seed stock was titrated by the haemagglutination test as per OIE protocol [25]. The allantoic cavities of 10-day-old SPF ECEs were inoculated with 0.2 ml of seed virus suspension (1:100 diluted in 0.01 M phosphate-buffered saline, pH 7.2) and incubated for 72 h at 37 °C followed by chilling at 4 °C overnight. The allantoic fluid was harvested, clarified by centrifugation and then stored at − 80 °C. The ten-fold serially diluted virus was inoculated in the allantoic cavity of SPF ECEs and the virus titer was estimated as the egg infective dose 50% per ml (EID_50_/ml).

### Plant extracts and antiviral drugs

Two plants, *O. sanctum* (commonly known as *Tulsi*) and *A. arabica* (commonly known as *Babul*), native to the Indian subcontinent were used in this study. The leaves of these plants were collected from Madhya Pradesh, India and identified by Dr. S.S. Ghoke through standard morphological and anatomical techniques. Plants were authenticated and voucher numbers for *O. sanctum* (Specimen#: vi p-1050313) and *A. arabica* (Specimen#: vi p-174629) were obtained. Crude extracts of these two plant leaves were prepared in hydro-methanol (1:1 dilution with water) by standard hot continuous extraction method using Soxhlet apparatus [26]. Two class compounds each in identified plants, *O. sanctum* (terpenoid and polyphenol) and *A. arabica* (flavonoid and polyphenol) were isolated and purified by ultraviolet–visible prep HPLC technique [27]. Antiviral drugs, Amantadine hydrochloride (Sigma, USA) and Oseltamivir (FLUVIR, Hetero Drugs Ltd., India), the currently approved drugs for influenza [28] were used as drug control and hydro-methanol as vehicle control group.

### Toxicity assay of plant extracts and antiviral drugs

To determine the maximum non-toxic concentration (MNTC) of all the extracts of *O. sanctum* (crude extract_*ocimum*_, terpenoid_*ocimum*_, polyphenol_*ocimum*_) and *A. arabica* (crude extract_*acacia*_, flavonoid_*acacia*_ and polyphenol_*acacia*_)_*,*_ the guidelines prescribed by the Organization for Economic Co-operation and Development (OECD) were followed [29]_*.*_ Average egg biomass of 10 SPF ECEs which included the embryo along with its allantoic sac and yolk was measured and the maximum dose for each of the herbal extract groups that could be used without inducing toxicity to the embryo was calculated as 135 mg/kg of egg biomass [29]. Afterwards, the MNTC was again assessed in the *in ovo* model. 0.1 ml of two-fold dilution of each of the plant extracts (200, 175, 150, 135, 100, 75, 50, 25, 10 and 5 mg/0.1 ml, diluted in extraction medium hydro-methanol) were inoculated into the allantoic cavity of 10-days-old SPF ECEs in triplicates. The SPF ECEs inoculated with 0.1 ml of hydro-methanol alone served as vehicle control. The MNTCs of amantadine and oseltamivir antiviral drugs were determined by inoculating 5.33, 8 and 16 μg/0.1 ml and 1.75, 2.66 and 5.25 μg/0.1 ml, respectively into the allantoic cavity of 10-days-old SPF ECEs separately in triplicates. All the inoculated SPF ECEs were incubated at 37 °C till hatching and were candled twice daily for checking the viability.

### Assessment of efficacy of herbal extracts against H9N2 virus using *in ovo* model

The antiviral activity of each plant crude extract and its isolated class compounds were assessed in three different formats viz; virucidal (dose-dependent), therapeutic (time-dependent) and prophylactic (dose-dependent).

### Dose-dependent virucidal activity

**Fig. 1:**
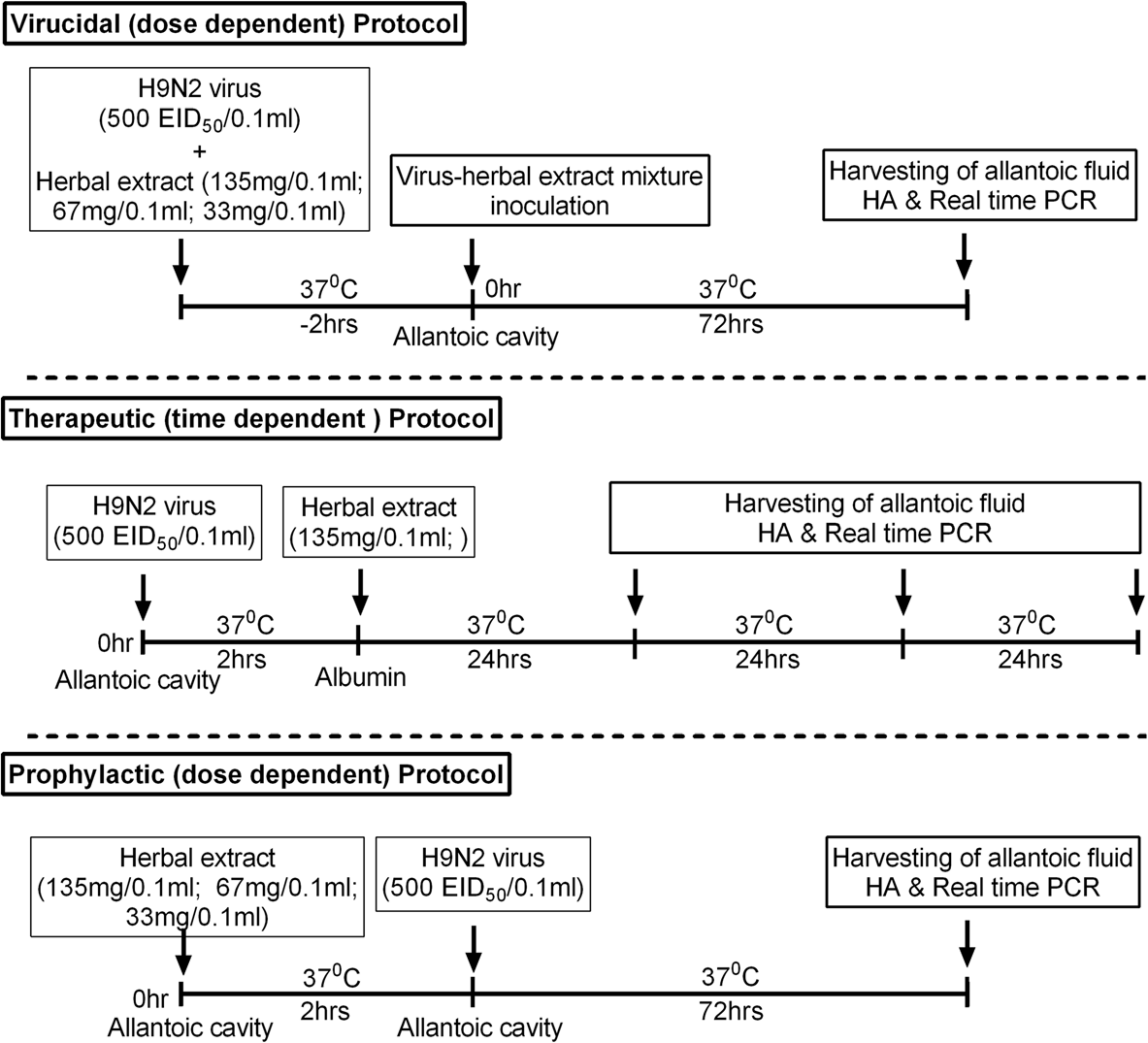
Experimental protocols for the assessment of the antiviral activity of *Ocimum sanctum* and *Acacia arabica* leave extracts against H9N2 virus using *in ovo* model

A total of nine treatment groups (each treatment group with five 10-days-old SPF ECEs) were made for the determination of virucidal activity of herbal extracts against A/chicken/CL/15–12/103075 (H9N2). Of the nine, six treatment groups corresponded to the respective six herbal extracts (crude extract_*ocimum*_, terpenoids_*ocimum*_, polyphenol_*ocimum*_; crude extract_*acacia*_, flavonoid_*acacia*_, polyphenol_*acacia*_) used in this study. The remaining three treatment groups were the drug control, the vehicle control and the virus control group. 0.1 ml of two-fold dilution of pre-calculated MNTC (135 mg, 67 mg, and 33 mg) of different plant leaves extracts (three extracts each from two different plants leaves) was incubated with 500 EID_50_/0.1 ml of H9N2 virus separately at 37 °C for 2 h. The drug control group included amantadine hydrochloride (MNTC-16 μg/0.1 ml) incubated with 500 EID_50_/0.1 ml of H9N2 virus and the virus control group included 500 EID_50_/0.1 ml of H9N2 virus incubated with 0.1 ml PBS for 2 h at 37 °C. The vehicle control group comprised of 0.1 ml of hydro-methanol incubated with 500 EID_50_/0.1 ml of H9N2 virus. Afterwards, 0.1 ml of each of the virus-extract mixture was inoculated into the allantoic cavity of 10-day-old SPF ECEs (5 each) separately and incubated at 37 °C for 72 h. The allantoic fluid was harvested from each of the treatment groups 72 h post-incubation. The presence of virus in the allantoic fluid was assessed by haemagglutination (HA) test as per methods described [30] and quantified by real time RT-qPCR [31] (Fig. 1).

### Therapeutic, time-dependent activity

Eight treatments groups (except the vehicle control group) as formulated for testing of virucidal antiviral activity were made for testing the therapeutic efficacy in a time dependent manner. In each of the treatment groups, 500 EID_50_/0.1 ml of H9N2 virus was inoculated into the allantoic cavity of 10-day-old SPF ECEs (6 eggs for each group) and incubated at 37 °C for 2 h. Then, the MNTC dose (135 mg/0.1 ml) of each plant extract was injected into the sharp pole of albumen of these ECEs separately and incubated at 37 °C. This route has been suggested as an improved model for testing antiviral drugs especially if the bioavailability of the drug is poor [32]. In the drug control group, oseltamivir (MNTC–2.66 μg/0.1 ml) was injected through albumen route 2 h post-virus inoculation. The allantoic fluid was harvested from each of the treatment group at 24, 48 and 72 h post-inoculation (Fig. 1). The presence of virus in the allantoic fluid was tested by HA test and quantified by real time RT-qPCR.

### Prophylactic, dose-dependent activity

For testing of prophylactic efficacy, same eight treatment groups as mentioned in above testing methodologies were used to examine the effect of extracts in a dose dependent manner. In each of the treatment groups, the two-fold dilution of MNTC (135 mg; 67 mg; 33 mg/0.1 ml) of different plant extracts (three extracts each from two plants leaves) was inoculated into the allantoic cavity of 10-day-old SPF ECEs (5 eggs for each group) and incubated at 37 °C for 2 h. Then, 500 EID_50_/0.1 ml of H9N2 virus was inoculated into the allantoic cavity of each of these eggs and incubated at 37 °C for 72 h (Fig. 1). In the drug control group, amantadine hydrochloride (MNTC-16 μg/0.1 ml) was given through allantoic cavity 2 h post-virus inoculation. The presence of virus in the allantoic fluid was detected by HA test and quantified by real time RT-qPCR.

### Real-time quantitative reverse transcription PCR

Viral RNA from the respective allantoic fluids was extracted using QIAamp Viral RNA mini kit (Qiagen, Germany) as per the recommendations of the manufacturer. One step RT-qPCR was carried out in duplicates as per standard method [31]. The PCR assay was conducted on Light Cycle® 480 Real-Time PCR, Roche, USA. The fluorescence reading was noted at the end of each extension step. No template control and no probe control were included in each run.

### Statistical analysis

The infectivity titer of the H9N2 virus (A/chicken/CL/15–12/103075) was expressed as EID_50_/ml and was calculated as per the standard method [33]. The data is presented as mean ± standard deviation. For the analysis of the significance of differences among the different treatment groups in terms of the virus genome copy number, two ways analysis of variance (ANOVA) with Tukey post hoc test was used. *P* values equal to or less than 0.05 were considered statistically significant. Analyses were performed using GraphPad Prism version 6.0 (GraphPad Software, SanDiego, CA, USA).

## Results

### Toxicity assay of plant extracts and antiviral drugs

In a first set of experiments, MNTC was calculated for each herbal extracts and antiviral drugs in the SPF ECEs. The criterion of non-toxicity of herbal extracts was identified as the absence of death of embryos up to hatching of all the eggs (three per drug concentration) at various concentrations (200, 175, 150, 135, 100, 75, 50, 25, 10 and 5 mg/0.1 ml). Irrespective of the different herbal extracts, all the ECEs that had received 200 mg/0.1 ml of extract died within 48 h of post-inoculation. The mortality of 50–15% was observed in ECEs given 175 and 150 mg/0.1 ml extract, while those that received 135 mg/0.1 ml or less showed no evidence of mortality till hatching. The concentrations of antiviral drugs tested were non-toxic to the embryos at the dose of 16 μg/0.1 ml and 2.66 μg/0.1 ml for amantadine hydrochloride and oseltamivir, respectively and thus have been used as MNTC throughout the study.

### Antiviral activity on the basis of haemagglutination test

The aim of this experiment was to identify the most effective extract in inhibiting the H9N2 virus replication in the SPF ECEs. The virus challenge dose was identified on the basis that there was no mortality in the eggs up to 72 h, in any of the virus challenge groups made, for evaluating the antiviral activities for different doses as well as time intervals.

#### Dose-dependent virucidal activity

**Fig. 2:**
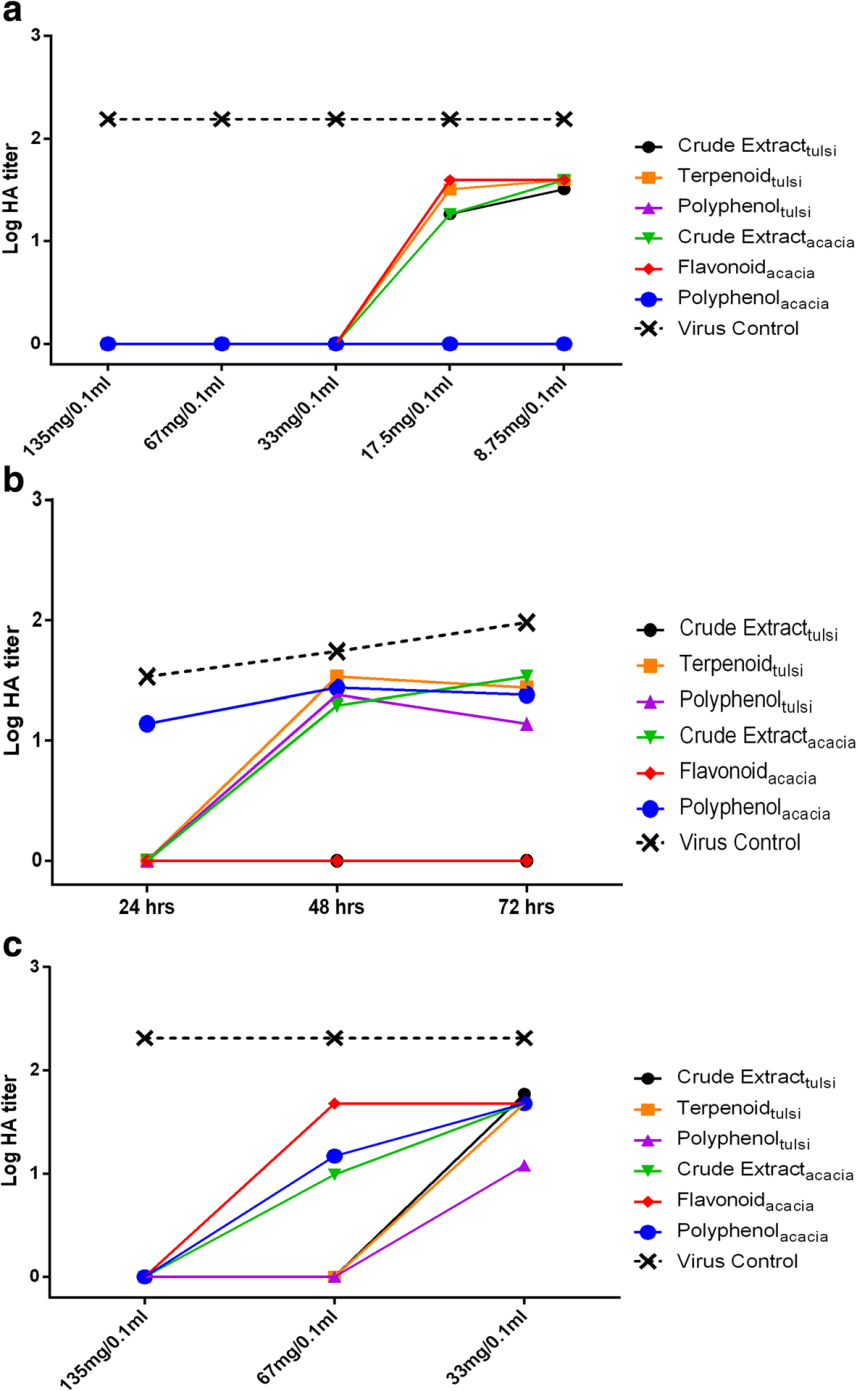
Measurement of antiviral activity of *O. sanctum* and *A. arabica* leave extracts against H9N2 virus using HA assay. **a** Dose-dependent virucidal activity; **b** Time-dependent therapeutic activity; **c** Dose-dependent prophylactic activity. The virus control group included 500 EID_50_/0.1 ml of H9N2 virus incubated with 0.1 ml PBS for 2 h at 37 °C. The HA titer were expressed as log HA titer

All the herbal extracts treatment groups including drug control did not show any HA titer irrespective of decreasing dose (135, 67, 33 mg/0.1 ml) treatments, indicating that even the low dose of all extracts were effective in inhibiting the replication of H9N2 virus. Allantoic fluid collected from these treatment groups were given two consecutive passages in SPF eggs to ascertain the absence of H9N2 replication which did not show any HA titer and reconfirmed the virucidal activity. However on further two-fold reduction in dose to 17.5 mg/0.1 ml for each extract, we found that crude extract_*ocimum*_, terpenoid_*ocimum*_, crude extract_*acacia*_ and flavonoid_*acacia*_ treatment groups showed a mean HA titer of 2^4.2^, 2^5^, 2^4.2^ and 2^5.3^, respectively which on further two fold reduction of dose to 8.75 mg/0.1 ml remained almost same as above indicating loss of virucidal activity at lower doses. However, polyphenol_*ocimum*_, polyphenol_*acacia*_ and the drug control amantadine treatment groups did not show any HA titer even at the lowest dose (8.75 mg/0.1 ml) tested indicating a high virucidal activity of these class compounds (Fig. 2).

#### Time-dependent therapeutic activity

Among the extracts of *O. sanctum*, only crude extract_*ocimum*_ treatment group showed absence of HA titer in the allantoic fluid harvested at all the three time intervals (24, 48 and 72 h) post-inoculation. The drug control group oseltamivir also showed absence of HA titer indicating no viral replication. The other two extract treatment groups, terpenoid_*ocimum*_ and polyphenol_*ocimum*,_ however had mean HA titers 2^4.8^ and 2^3.8^, respectively at 72 h post-inoculation (Fig. 2).

#### Dose-dependent prophylactic activity

All the three extracts of *O. sanctum* at doses 135 and 67 mg/0.1 ml showed prophylactic potential as revealed by the absence of HA titers. However, on further reduction of dose to 33 mg/0.1 ml, all the three extracts (crude extract_*ocimum*_, terpenoid_*ocimum*_ and polyphenol_*ocimum*_) showed a mean HA titer of 2^5.9^, 2^5.6^ and 2^3.6^ respectively. In the case of *A. arabica*, MNTC dose (135 mg/0.1 ml) of all the three extracts showed absence of HA titer. Lower doses (67 and 33 mg/0.1 ml) of any of the extracts of *A. arabica* were not effective in preventing viral replication. The drug control group showed complete absence of HA titer (Fig. 2).

### Antiviral activity on the basis of viral quantification by real time RT-qPCR

#### Virucidal, dose-dependent activity

**Fig. 3:**
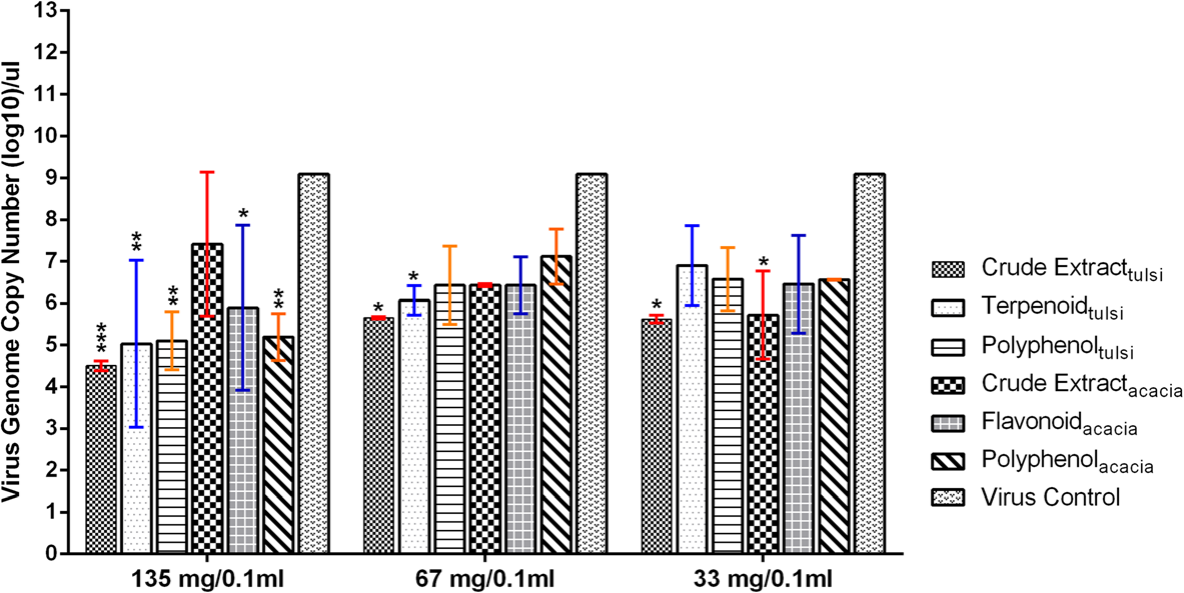
Assessment of virucidal activity of the *O. sanctum* and *A. arabica* leaves extracts in a dose-dependent manner by Real Time RT-qPCR assay. Each error bar represents standard deviation (SD) from the 5 ECEs. The virus control group included 500 EID_50_/0.1 ml of H9N2 virus. Statistical significance was assessed by two ways analysis of variance (ANOVA) with Tukey post-hoc test (*p < 0.05, **p < 0.01, ***p < 0.001, ****p < 0.0001)

The aim of this experiment was to quantify the viral amount and identify the most effective extract in inhibiting the H9N2 virus replication. The crude extract_*ocimum*_ when administered at the dose of 135 mg/0.1 ml significantly inhibited the virus replication compared to virus control group (*p* < 0.001). At the same dose (135 mg/0.1 ml), the other four extracts; terpenoid_*ocimum*_, polyphenol_*ocimum*_, flavonoid_acacia_ and polyphenol_acacia_ also inhibited the virus replication significantly (*p* < 0.05–0.01) (Fig. 3). On reducing the dose (67 mg/0.1 ml), only crude extract_*ocimum*_ and terpenoid_*ocimum*_ were effective (*p* < 0.05). Further reducing the dose (33 mg/0.1 ml), crude extract_*ocimum*_ remained effective (*p* < 0.05) (Fig. 3). Non-significant efficacy was recorded within both groups of herbal extracts of *O. sanctum* and *A. arabica* in the present study.

##### Therapeutic, time-dependent activity

**Fig. 4:**
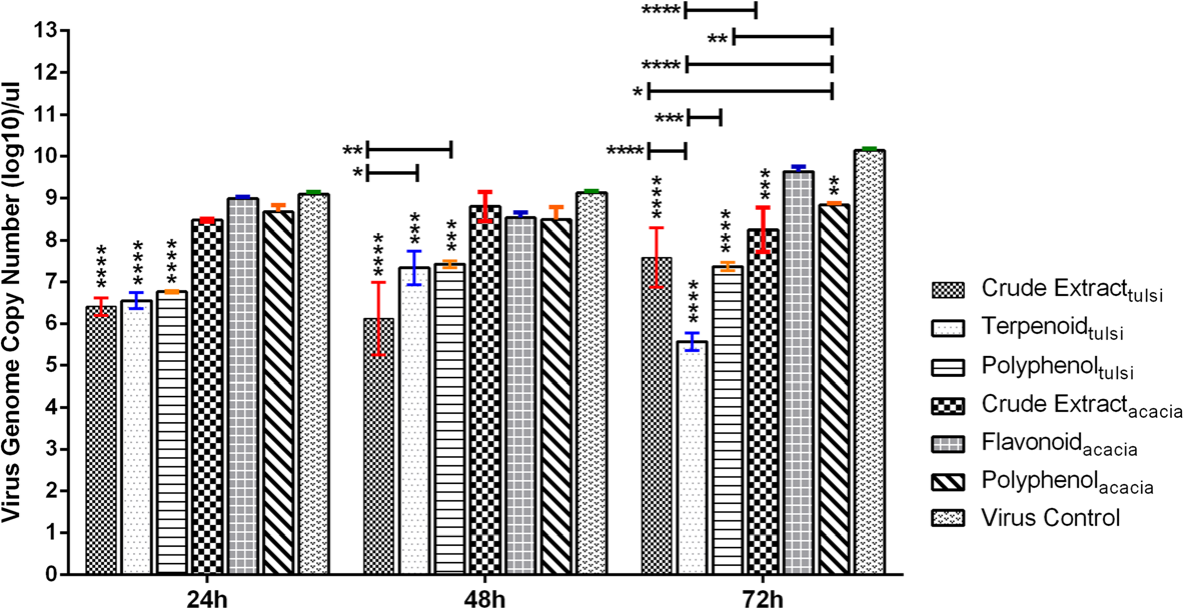
Assessment of therapeutic activity of the *O. sanctum* and *A. arabica* leaves extracts in a time-dependent manner by Real Time RT-qPCR assay. Each error bar represents standard deviation (SD) from the 6 ECEs. The virus control group included 500 EID_50_/0.1 ml of H9N2 virus. Statistical significance was assessed by two ways analysis of variance (ANOVA) with Tukey post-hoc test (*p < 0.05, **p < 0.01, ***p < 0.001, ****p < 0.0001)

All the three extracts of *O. sanctum* (crude extract_*ocimum*_, terpenoid_*ocimum*_ and polyphenol_*ocimum*_) were significantly effective in limiting the H9N2 virus replication in the SPF ECEs at different time intervals (24, 48 and 72 h post-inoculation) (*p* < 0.001–0.0001). Within the *O. sanctum* treatment group, crude extract_*ocimum*_ was found to have significantly high efficacy in comparison to terpenoid_*ocimum*_ (*p* < 0.05) and polyphenol_*ocimum*_ (*p* < 0.01) at 48 h post-inoculation as indicated by decrease in viral genome copy numbers (Fig. 4). However, terpenoid_*ocimum*_ showed significantly high inhibitory activity compared to crude extract_*ocimum*_ (*p* < 0.0001) and polyphenol_*ocimum*_ (*p* < 0.001) at 72 h post-inoculation.

In the case of *A. Arabica*, none of the extracts were effective initially at 24 and 48 h post-inoculation (*p* > 0.05), however, at 72 h post-inoculation, crude extract_*acacia*_ (*p* < 0.001) and polyphenol_*acacia*_ (*p* < 0.01) depicted therapeutic activity as indicated by significant decrease in the virus genome copy numbers as compared to virus control group (Fig. 4). However, differences in the time dependent therapeutic effect among the extracts of *A. arabica* were non-significant (*p* > 0.05). On comparison of antiviral treatment between the two groups it was found that all three extracts of *O. sanctum* treatment group had significantly higher (*p* < 0.05–0.0001) antiviral efficacy than the crude extract_*acasia*_ or polyphenol_*acacia*_ extracts.

##### Prophylactic, dose-dependent activity

**Fig. 5:**
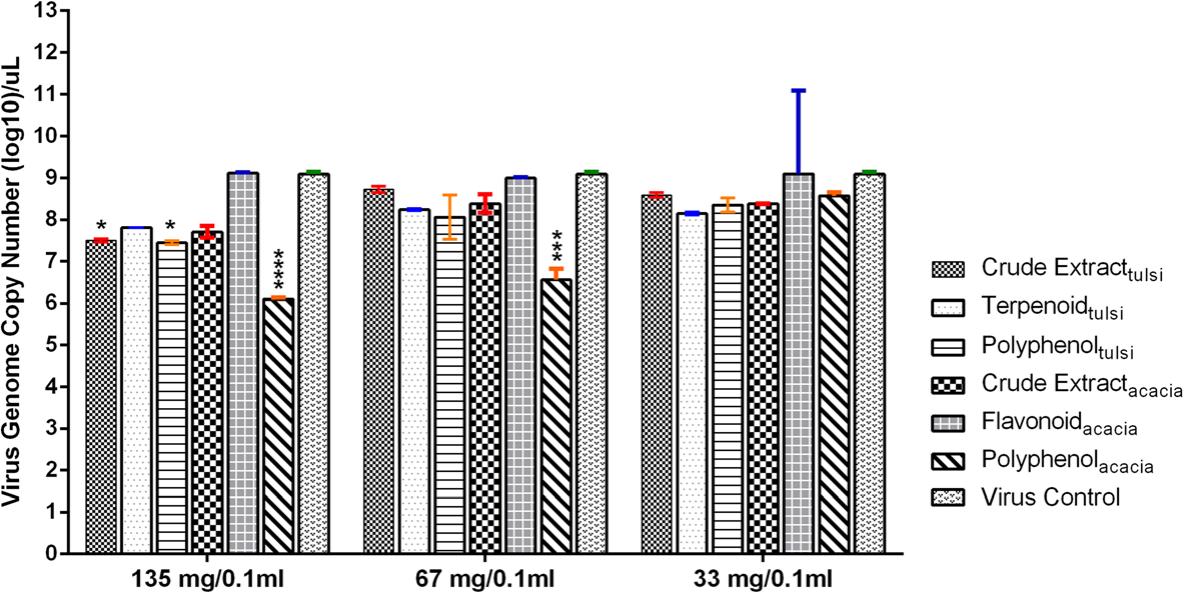
Assessment of prophylactic activity of the *O. sanctum* and *A. arabica* leaves extracts in a dose-dependent manner by Real Time RT-qPCR assay. Each error bar represents standard deviation (SD) from the 5 ECEs. The virus control group included 500 EID_50_/0.1 ml of H9N2 virus. Statistical significance was assessed by two ways analysis of variance (ANOVA) with Tukey post-hoc test (*p < 0.05, **p < 0.01, ***p < 0.001, ****p < 0.0001)

The prophylactic potential of all the herbal extracts were determined against H9N2 virus in the SPF ECEs. In the *O. sanctum* group, only crude extract_*ocimum*_ and polyphenol_*ocimum*_ treated ECEs showed significant (*p* < 0.05) decrease in viral genome copy numbers at dose of 135 mg/0.1 ml (Fig. 5). The decrease in viral genome copy numbers was non-significant at lower doses of 67 and 33 mg/0.1 ml in all the three extracts of *O. sanctum* groups. In *A. arabica* group, only polyphenol_*acacia*_ was more successful in reducing the viral genome copy numbers at doses of 135 (*p* < 0.0001) and 67 mg/0.1 ml (*p* < 0.001) while it became ineffective at low dose of 33 mg/0.1 ml (*p* > 0.05) (Fig. 5). The other two extracts of *A. arabica*, crude extract_*acacia*_ and flavonoid_*acacia*_ were not effective as prophylactic extract at any of the doses (*p* > 0.05).

## Discussion

Spread of H9N2 subtype has demonstrated an increased theoretical threat to humans because of the potential emergence of novel subtypes of avian influenza [13]. It is being considered as an emerging pandemic threat in view of the clinical and confirmed cases of H9N2 in China, Hong Kong, Bangladesh and Egypt [14]. This reaffirms the need for search of new compounds with antiviral activity. With increase in drug resistance to synthetic antivirals, natural products remain an important alternative for the control infectious diseases. In the present study, the *in ovo* model has been used for studying antiviral activities of different leaves extracts of *Ocimum sanctum* and *Acacia arabica* against low pathogenic avian influenza (LPAI) H9N2.

In our previous work we were able to demonstrate 100% virucidal activity of cold aqueous extract of bark of *A. arabica* at a concentration of 0.625 mg/ml against HPAI H5N1 using in vitro model. Mild therapeutic activity was also demonstrated up to 48 h in cold aqueous extract of *Ocimum tenuiflorum* when tested against HPAI H5N1 [20].

In the present work, we had selected two plants, *Ocimum sanctum* and *Acacia arabica* for studying their efficacy against avian influenza H9N2 virus because these plants are widely distributed and easily available throughout various geographical locations in India and have strong record in the literature for their antiviral activity against different viruses [16, 17]. Three different approaches viz. virucidal (dose dependent), therapeutic (time dependent) and prophylactic (dose dependent) were employed to systematically explore the potential of extracts of leaves of *O. sanctum* (crude extract, terpenoid and polyphenol) and *A. arabica* (crude extract, flavonoid and polyphenol) against LPAI H9N2 using *in ovo* model.

In dose dependent virucidal activity, all the extracts of both the plants in doses (135, 67 and 33 mg/0.1 ml) displayed absence of HA titer indicating that all the extracts had inhibitory potential. On further reduction of dose rate in double dilution, polyphenol_acasia_ and polyphenol _Ocimum_ only maintained virucidal activity up to a low dose rate of 8.75 mg/0.1 ml which was confirmed by absence of HA in the harvested allantoic fluid of ECEs in the next two passages also. This virucidal effect could be due to masking/blocking of HA protein of the virus by the herbal extracts during the incubation period, which might have inhibited the H9N2 virus replication. Catechin compounds in polyphenols are known to have antiviral activity mediated by preventing adsorption of viruses to cells [34, 35]. Highly significant to significant decrease in the viral genome copy numbers was recorded in all the extracts at the MNTC dose (135 mg/0.1 ml) except crude extract_*acacia*_ (*P* < 0.05–0.001). Although the virucidal effect was seen more prominently in the HA test where the virus HA titer was absent in comparison to the virus control (mean HA titer 2^7.3^) and vehicle control (mean HA titer 2^5.8^), the quantification of the virus by real time RT-qPCR also corroborated with the HA results especially at higher extract doses. The results of both the assays clearly indicated that crude extract_ocimum_ in particular is a potent inhibitor of H9N2 virus replication.

Our study is further supported by the fact that individual plant extracts were less effective compared to crude extracts which may due to synergistic effect of individual component and some of the uncharacterised components [36]. Earlier studies for evaluation of virucidal activity against H9N2 virus by crude extract_*echinecea*_ and crude extract_*sambucus*_ against H9N2 virus has also been ascribed to blocking of the influenza virus entry via at least two virion targets, HA and NA on the surface of virus [37]. Mentofin® (combination of eucalyptus oil and peppermint) in another study has been reported to have complete virucidal activity against H9N2 virus in the presence of organic matter (skimmed milk) [38].

On assessing the therapeutic potential, we were able to demonstrate that all the extracts of *O. sanctum* (crude extract_*ocimum*_, terpenoid_*ocimum*_ and polyphenol_*ocimum*_) significantly reduced the viral genome copy numbers (*P* < 0.0001) at all the tested time intervals of 24, 48 and 72 h post-inoculation. However, crude extract_*ocimum*_ and terpenoid_*ocimum*_ had higher therapeutic potential at 48 and 72 h post-inoculation, respectively. Although terpenoid_*ocimum*_ and polyphenol_*ocimum*_ treated groups showed a mean HA titer of 2^5.1^, 2^4.8^, respectively at 48 h post-inoculation and a slightly decreased mean HA titer of 2^4.8^, 2^3.8^, respectively at 72 h post-inoculation, the genome copy numbers in these treated groups were significantly reduced in comparison to virus control (*P* < 0.0001). The possible reason for this could be due to the decrease in concentration of the active ingredient which might have allowed the virus to replicate because of single dose regimen followed in the experiment. In addition, other possible reason might be the high sensitivity of real time PCR assay. Surprisingly, in *A. arabica* treated groups, crude extract_*acacia*_ and polyphenol_*acacia*_ showed significant decrease in viral genome copy numbers at a later time interval of 72 h post-inoculation (*P* < 0.01). Since the route of administration was via albumen, slow movement/distribution of *A. arabica* extracts from albumen to allantoic cavity might be the reason for delayed effect. Variable bioavailability of drugs, oseltamivir and ribavirin via albumin route has been established previously also [39].

In the case of dose dependent prophylactic activity assessment, only the high doses of crude extract_*ocimum*_, polyphenol_*ocimum*_ and polyphenol_*acacia*_ were effective as observed by the absence of HA titer and significant decrease in viral genome copy numbers. Lower doses of these extracts failed to provide sufficient prophylactic activity. Activity of different polyphenolic compounds in plants worked their best when added to the cells just around or before the time of virus adsorption using in vitro model. They do possess high binding affinities with viral HA and NA which might be a reason for their prophylactic activity [34, 40–42]. Thus, the therapeutic or prophylactic efficacy testing using *in ovo* model could be improved by considering the multiple dose regimens and monitoring the active ingredients diffusion of plant extracts through suitable methods. Moreover, since these extracts shows no adverse effect, a clinical pilot study would give further information on the potency of these extracts in protecting against H9N2 virus infection in chickens.

## Conclusions

The treatment with the crude extract derived from the leaves of *Ocimum sanctum* leads to the significant H9N2 virus reduction in assessing the all the three; virucidal, therapeutic and prophylactic activities using *in ovo* model. At this stage, we conclude that crude extract_*ocimum*_ could be a promising extract for developing safe and efficacious antiviral compound against H9N2 virus. The protective efficacy of crude extract_*ocimum*_ might be attributed to multiple mechanisms of action, e.g. specific inhibition of a stage in viral intracellular multiplication and non-specific interference with virus-cell interactions like masking/blocking the HA glycoprotein. Furthermore, terpenoid_*ocimum*_ was also effective for virucidal and therapeutic activity and polyphenol_*acacia*_ for prophylactic activity. Nevertheless, it would of great importance to study further which particular active ingredient or a combination of these three compounds (crude extract_*ocimum*_, terpenoid_*ocimum*_ and polyphenol_*acacia*_) could provide broader antiviral activity against H9N2 virus. This study also established the use of influenza virus-infected chick embryos for evaluation of new antiviral substances which could reduce the use of small laboratory animals required for such studies.

## Abbreviations

ECE: Embryonated chicken egg
EID_50_: Egg infectious dose 50%
HA: Haemagglutination
HI: Haemagglutination inhibition
HPAI: Highly pathogenic avian influenza
LPAI: Low pathogenic avian influenza
MNTC: Maximum non-toxic concentration
OECD: Organization for Economic Co-operation and Development
PBS: Phosphate buffer saline
RT-qPCR: Quantitative reverse transcription polymerase chain reaction
SPF: Specific pathogen free
